# Side Gliding With Untrained External Assistance and Preexisting Hip Internal Rotation Weakness Preceding Acute Lateral Hip Pain: A Mechanical Diagnosis and Therapy (MDT) Case Report

**DOI:** 10.7759/cureus.109892

**Published:** 2026-05-29

**Authors:** Thomas N Fairbank

**Affiliations:** 1 Physical Therapy, Texas Health Back Care (Spine Team Texas), Rockwall, USA

**Keywords:** adverse event, low back, lower extremity radiculopathy, lumbar spine pain, mckenzie method, mechanical diagnosis and therapy, physical therapy

## Abstract

A patient complained of left-sided low back pain that radiated to her left ankle and, upon examination, was found to have right hip internal rotation weakness. She was classified by this author as having a directional preference for lumbar rotation in flexion per the McKenzie Method of Mechanical Diagnosis and Therapy (MDT, or the McKenzie Method). At the patient's eighth visit, she reported that she had been performing approximately 160 repetitions of left lumbar side gliding against a wall per day and, of her own volition, having an untrained family member give this movement a push. At this visit, manual correction of a lateral lumbar shift, also known as a shift correction, slightly improved her lumbar active range of motion (AROM). The clinician then asked the patient to perform left lumbar side gliding against a wall in the clinic during which a “pop” was heard, and the patient reported intense right hip pain as well as displayed an antalgic gait. Despite numerous limitations including inadequate documentation of subjective and objective baselines, such as numeric pain rating scale, strength, and AROM immediately after this critical event as well as multiple confounding factors, it is possible that future clinicians may wish to utilize the shift correction manual technique, instead of additional repetitions of lumbar side gliding, to more accurately determine directional preference.

## Introduction

Despite the popularity of the McKenzie Method of Mechanical Diagnosis and Therapy (MDT, or the McKenzie method) [1,2], to this author's knowledge, no adverse events using this intervention have been reported [3]. This method involves assessing the effect of different movements to determine whether a patient has a directional preference or non-directional preference classification. Systematic reviews describe empirical support for the concept of direction preference with mixed effect sizes regarding efficacy, while endorsing the safety of this approach [4-12]. The safety of this method may be due in part to the use of force progression, which means that typically patient-generated forces are used before clinician-generated forces and that mobilizations are utilized prior to manipulation [13]. Despite the treating clinician utilizing the force progression concept of MDT, this patient experienced an adverse event in the context of performing around 160 repetitions of lumbar side gliding against a wall per day and, of her own volition, having an untrained family member provide external force to side gliding. Additionally, at her initial examination, she was found to have right hip internal rotation weakness, which may have made her more susceptible to an adverse event of acute right hip pain. However, this case report has numerous limitations including inadequate documentation of subjective and objective baselines, such as NPRS (numeric pain rating scale), strength, and AROM (active range of motion) immediately after this critical event.

## Case presentation

The patient was a 61-year-old woman who presented to Physical Therapy with complaints of left low back and left leg pain, from her hip distal to her ankle. She was a school district employee with a job that sometimes involved lifting young children. Her medical history was noteworthy only for scoliosis. No red flags were present. The patient did not have osteoporosis, osteopenia, prior hip joint pathology, or a history of prolonged corticosteroid use.

Symptoms were intermittent and had been present for two years. Symptoms would occur with walking, sleeping on her side, and getting in or out of a car. Her FOTO score was 57/100, which indicates moderate difficulty performing usual activities [14]. She reported her pain intensity was 10/10 at its worst. She reported slight improvement since taking prednisone somewhat recently.

The patient appeared to have a right lateral shift deformity, in which her trunk and shoulders were positioned to the right in relation to her pelvis, and the patient was unable to correct this shift. She presented with a painful and severe loss of left lumbar side gliding and a pain-free but moderate loss of lumbar flexion and extension. Right side gliding was pain-free and full. A limitation of this case report is the lack of numeric quantification of lumbar AROM.

Manual muscle testing revealed 5/5 strength for all lower extremity muscles, except the following: hip abduction: 4/5 R (pain-free) and 3-/5 L (with pain); hip internal rotation 3-/5 R and 5/5 L (her right side was weaker, but the left was her symptomatic side)

Differential diagnosis

Per MDT classification, the patient was assessed to see if she presented with a lumbar derangement, also known as having a directional preference in which a specific direction of movement reduces symptoms and improves baselines, such as AROM. This assessment can also classify a patient as having a non-derangement pathology [15], which would include an inflammatory condition, spinal stenosis, spinal tumor, hip pathology, sacroiliac joint dysfunction, and central sensitization. The treating physical therapist was an orthopedic clinical specialist (OCS), a Fellow in the American Academy of Orthopedic Manual Physical Therapists (FAAOMPT), and a diplomat in the McKenzie Method (Dip. MDT).

**Figure 1:**
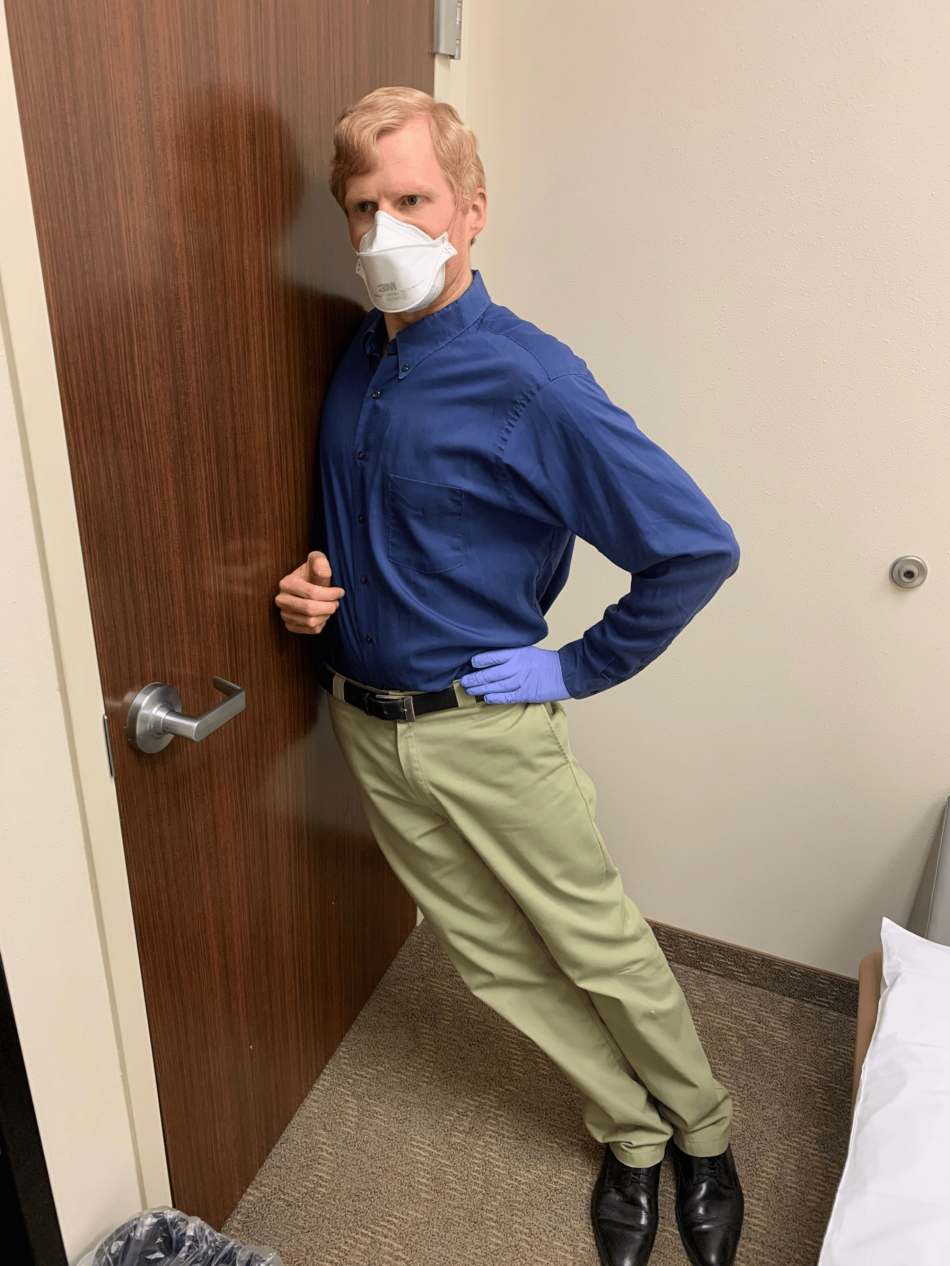
Left lumbar side gliding against a wall. Representative image depicting the treating physical therapist.

**Figure 2:**
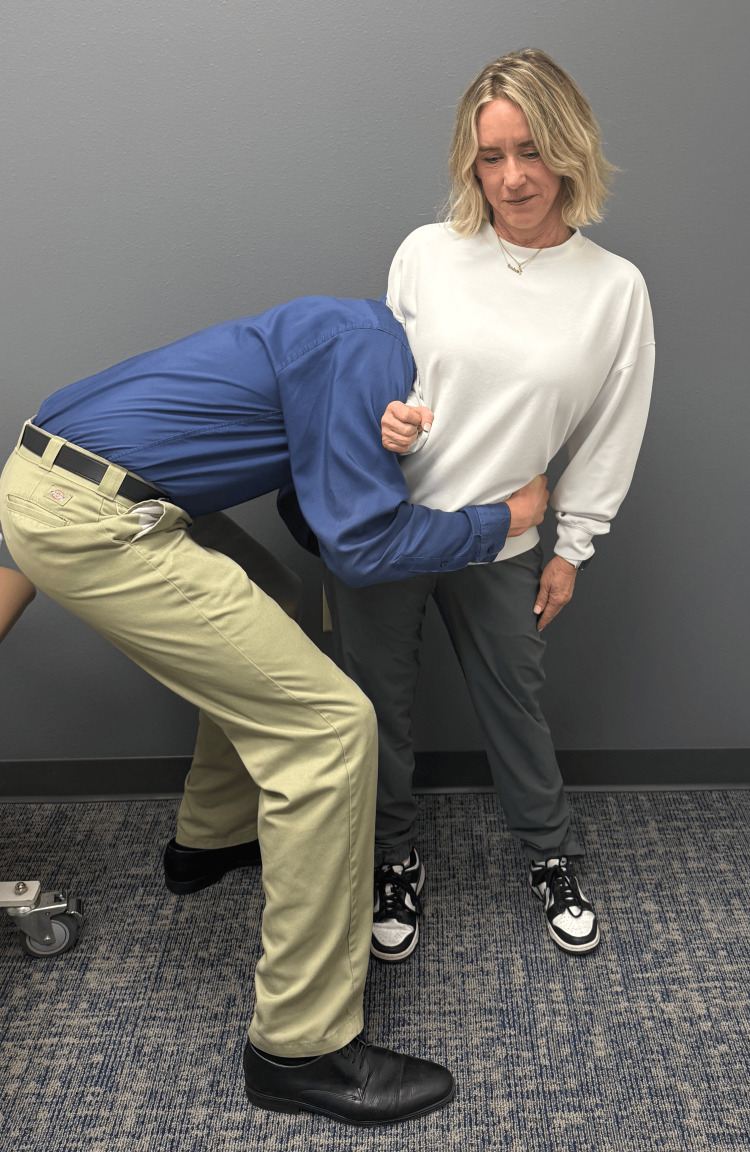
Manual correction of a lateral lumbar shift also known as shift correction. Shown here for left lumbar side gliding. Representative image using a human model. Photograph posed by a model and does not depict the patient described in this case report.

Left lumbar side gliding was attempted both against a wall (Figure 1) and manual correction of a lateral lumbar shift, also known as shift correction (Figure 2). The shift correction performed was just four repetitions of mid-range because the patient found this movement very painful. Afterwards, she was worse, with more pain in standing.

**Figure 3:**
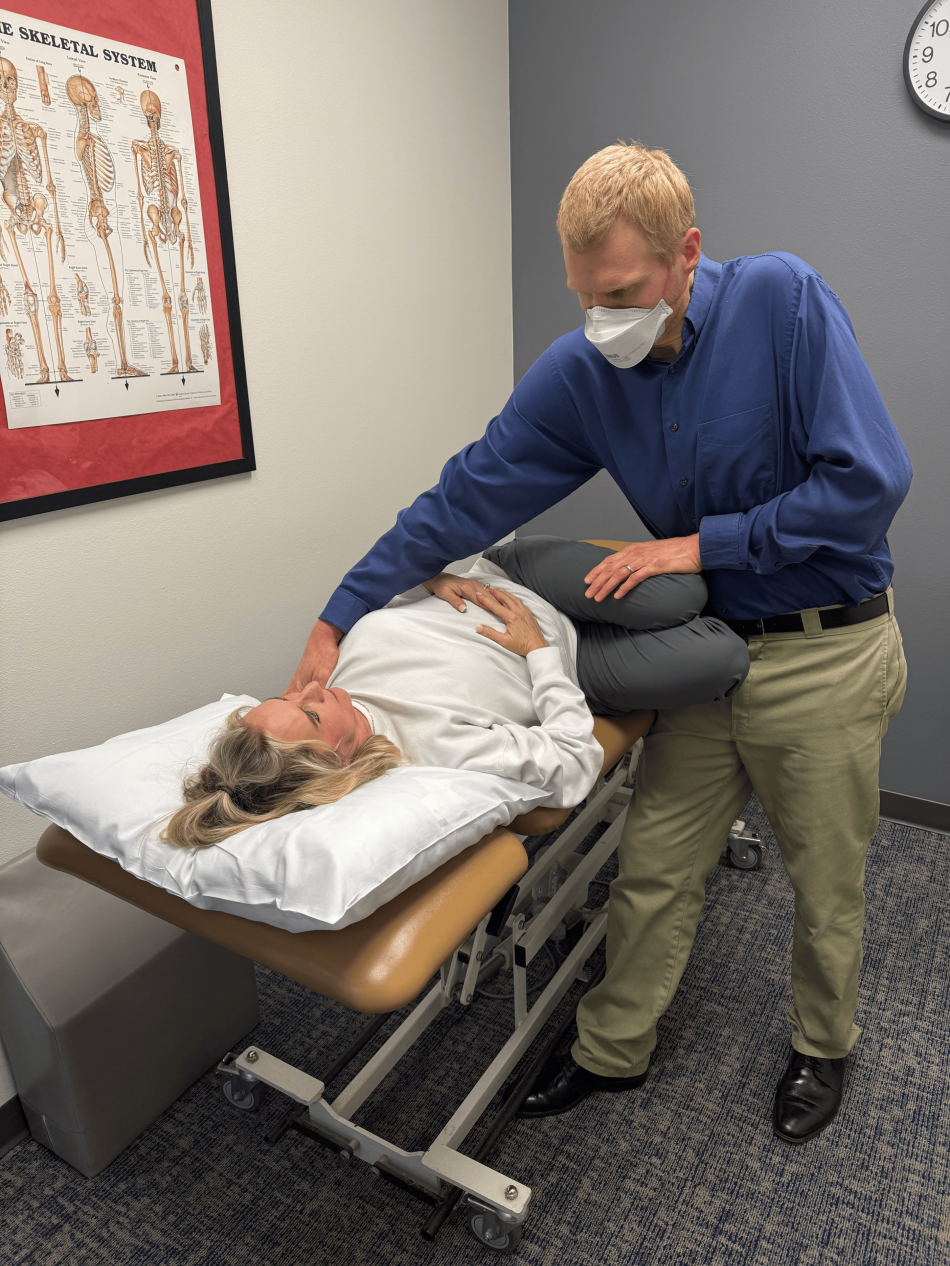
Lumbar rotation in flexion shown here for left rotation. Representative image using a human model. Photograph posed by a model and does not depict the patient described in this case report.

**Figure 4:**
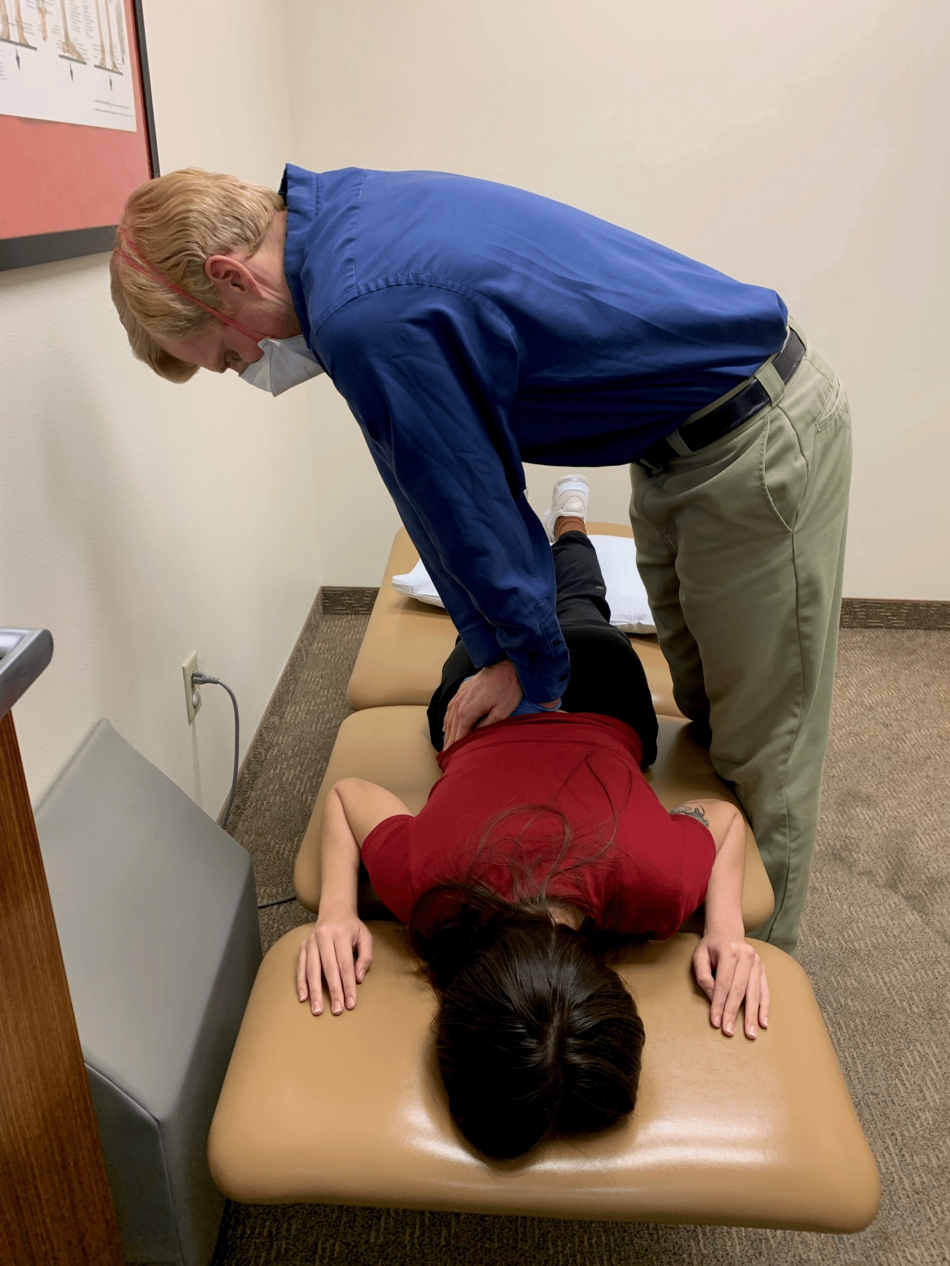
Lumbar rotation mobilization in extension shown here on the right side of the spine. Representative image using a human model. Photograph posed by a model and does not depict the patient described in this case report.

She was better after lumbar rotation in flexion (Figure 3) with more lumbar AROM and less pain. More improvement was noted after left lumbar rotation in flexion than right. Her response was even better after lumbar rotation mobilization in extension (Figure 4).

The patient was classified as having a lumbar derangement because of her rapid improvement. She was instructed to perform left lumbar rotation in flexion every two hours for several days until her next follow-up visit.

Treatment

At visits two through five, over a period of roughly three weeks, the patient continued to report adherence to her home program, but no change to her functional mobility. Various MDT movements were attempted, which all either worsened her presentation or had no effect. During visit three, left lumbar rotation in flexion worsened the patient's pain (Table 1). At this visit, she was instructed to perform left lumbar side gliding against a wall, which produced her pain, but afterwards, she was no worse.

**Table 1:** Description of response to MDT movements by visit number. MDT: The McKenzie Method of Mechanical Diagnosis and Therapy

Visit Number	MDT Movement	Overall effect	Home Program
Visit One	Shift correction for left lumbar side gliding	Worse	
	Left lumbar rotation in flexion	Better	Left lumbar rotation in flexion
Visit Three	Left lumbar rotation in flexion	Worse	
	Left lumbar side gliding against a wall	Produce pain, no worse after	Left lumbar side gliding against a wall
Visit Four	Shift correction for left lumbar side gliding	Worse	
	Right lumbar rotation in flexion	Better	Right lumbar rotation in flexion
Visit Five	Right lumbar rotation in flexion followed by repeated extension in lying	Better	Right lumbar rotation in flexion followed by repeated extension in lying

On visit four, the patient was objectively better, in that her left lumbar side gliding range of motion had improved to be pain-free, with now only a moderate loss. Shift correction in this direction resulted in her being worse afterwards. At this visit, the patient was instructed to perform right lumbar rotation in flexion as her new home program, as this now produced a better response, with more AROM and less pain with movement afterwards (Table 1).

At visit five, she was told to continue performing right lumbar rotation in flexion, followed by repeated extension in lying, which reduced her pain with walking (Table 1).

At visit six, two weeks later, she stated that she was much better and no longer had pain with sleeping on her side, getting in or out of a car, or walking. She said the only time she had pain now was when her dog pulled her, and this was an intensity of 4/10 at its worst. Her FOTO score had improved by 31 points, to 88/100, which is greater than the minimal clinically important improvement of five [14]. Beyond this, she now displayed full, pain-free, lumbar AROM into extension, flexion, and bilateral side gliding. The clinician anticipated two more visits until discharge. The patient was instructed to continue with the same directional preference exercises she had been performing since her last visit, which were right lumbar rotation in flexion, followed by repeated extension in lying.

At her seventh visit, nine days later, the patient reported she still sometimes had pain with walking, but now she knew how to get rid of it. The patient and treating clinician agreed that her next visit would be her last.

Outcome

After 18 more days, she attended her eighth visit. At the start of this visit, she reported that her pain had increased in the past week for no apparent reason. The pain was located primarily in her left leg, and a few brief times while sitting, her entire right leg, from her hip down to her ankle, felt like “lightning.” During the past 18 days, she had performed her home program of right lumbar rotation in flexion only about three times a day, less often than the nine times a day that were recommended.

Over the past 18 days, she had also been performing left lumbar side gliding against a wall roughly eight times a day. She would even have her granddaughter push to increase this stretch. It is unclear whether her granddaughter was performing shift correction or pushing in some other manner. Having a family member give this exercise a push was not a recommendation by the treating clinician; instead, it was a result of the patient's own volition. She reported that left lumbar side gliding against a wall would abolish her left leg pain. Furthermore, she stated that in the morning, she could visibly see her hips were not level, and left lumbar side gliding against a wall would correct this visual asymmetry.

At this visit, the main objective finding was that lumbar flexion was painful, which was not present in any prior visit. To help clarify why the patient was worse since her last visit, the clinician asked the patient to perform one to two sets of left lumbar side gliding against a wall. This had no effect on her lumbar AROM. At this point, the treating clinician performed manual correction of a lateral lumbar shift, also known as a shift correction, in this same direction, which slightly improved her lumbar AROM, although her pain with lumbar flexion was unchanged. Then the clinician asked the patient to perform another set of 10-20 repetitions of left lumbar side gliding against a wall. After about eight repetitions, while still in the clinic, an audible “pop” was heard. She reported intense pain at her right greater trochanter and displayed an antalgic gait. This new pain was in her right hip, which is the hip that is closer to the wall with this exercise (Figure 1). The initial pain that brought her to therapy was on her left side. Of note, one of the numerous limitations of this study is the lack of subjective and objective information available after this critical event.

She went to Urgent Care, and an X-ray found “minimal enthesopathy along the right greater trochanter”. The enthesis is where tendons, ligaments, and other soft tissues attach to bone [16]. The provider at Urgent Care told her to take the next two and a half days off from work. The treating Physical Therapist called the patient three days later. She said she was back at work, it was going well, but she was still having right hip pain, particularly with stair navigation, and she reported bruising on her right hip.

Several weeks later, the patient saw an orthopedic doctor who sent the patient to a different Physical Therapist for one visit to learn a home program. The patient reported to the original treating clinician via phone that she thought her care was backwards; she would have preferred having imaging prior to starting Physical Therapy, so that these tests could have been used by her original Physical Therapist to guide treatment. Despite this patient's perspective, it is important to note that a considerable body of research endorses Physical Therapy prior to imaging for the vast majority of patients, and it has been found that sometimes early imaging can be detrimental to patients [17].

## Discussion

Side gliding is a safe and effective exercise for many patients [4-12], and this single case observed a temporal association between side gliding against a wall and acute hip pain. One case cannot determine causality. Rather, it is possible that side gliding may have been a contributing factor to the acute hip pain. Multiple confounders exist; this case involved about 160 repetitions per day of lumbar side gliding against a wall for a period of 18 days, an untrained external force being applied to side gliding by a family member, and also preexisting right hip internal rotation weakness, which may have made her more susceptible to an adverse event of acute right hip pain. These factors limit any causal inference that side gliding was the sole cause. Instead, this case is best framed as hypothesis-generating.

After the acute hip pain, the patient's X-ray findings were of minimal enthesopathy, yet in many cases, enthesopathies are asymptomatic [18]. Furthermore, clinical practice guidelines recommend against the use of radiographs to diagnose enthesopathy, due to their limited accuracy [19], which calls into question whether the acute right hip pain was due to an enthesopathy. Additionally, in the initial evaluation, the patient displayed limited right hip internal rotation strength, suggesting that an enthesopathy may have been present prior to performing side gliding against a wall. Beyond this, radiographs are far more sensitive at detecting chronic enthesopathies, rather than acute enthesopathies [19], which increases the likelihood that the enthesopathy found on X-ray was a preexisting condition.

Regardless, it is plausible that only those individuals with hip weakness may be at risk of this type of adverse event. The audible “pop” may represent an acute tendon event (gluteus medius/minimus overload/partial tear) or trochanteric snapping. Advanced imaging such as an ultrasound or MRI was not performed, which may have provided additional information on the mechanism. In addition to tendon degeneration being at least partially responsible for the acute hip pain, other possible explanations include referred pain from her spine, unobserved trauma, and normal variability.

It is possible that this type of critical event can only occur if one has been performing multiple sets of side gliding with an external force being applied by an untrained individual. This patient, of her own volition, had her granddaughter push to increase the stretch of side gliding between her seventh and eighth visits; it is unknown in what way her granddaughter was giving this movement a push.

Given the patient's response to left lumbar side gliding, it appears the initial hypothesis that the patient had a right lateral shift deformity was mistaken. Instead, it is likely that the patient's scoliosis caused her spine to curve in this direction. On the patient's eighth visit, shift correction did not result in a significant improvement in baselines, which suggests that left lumbar side gliding was not sustained as a directional preference and that additional repetitions of side gliding against a wall did not need to be performed at that time in the clinic where the critical event occurred [20].

**Figure 5:**
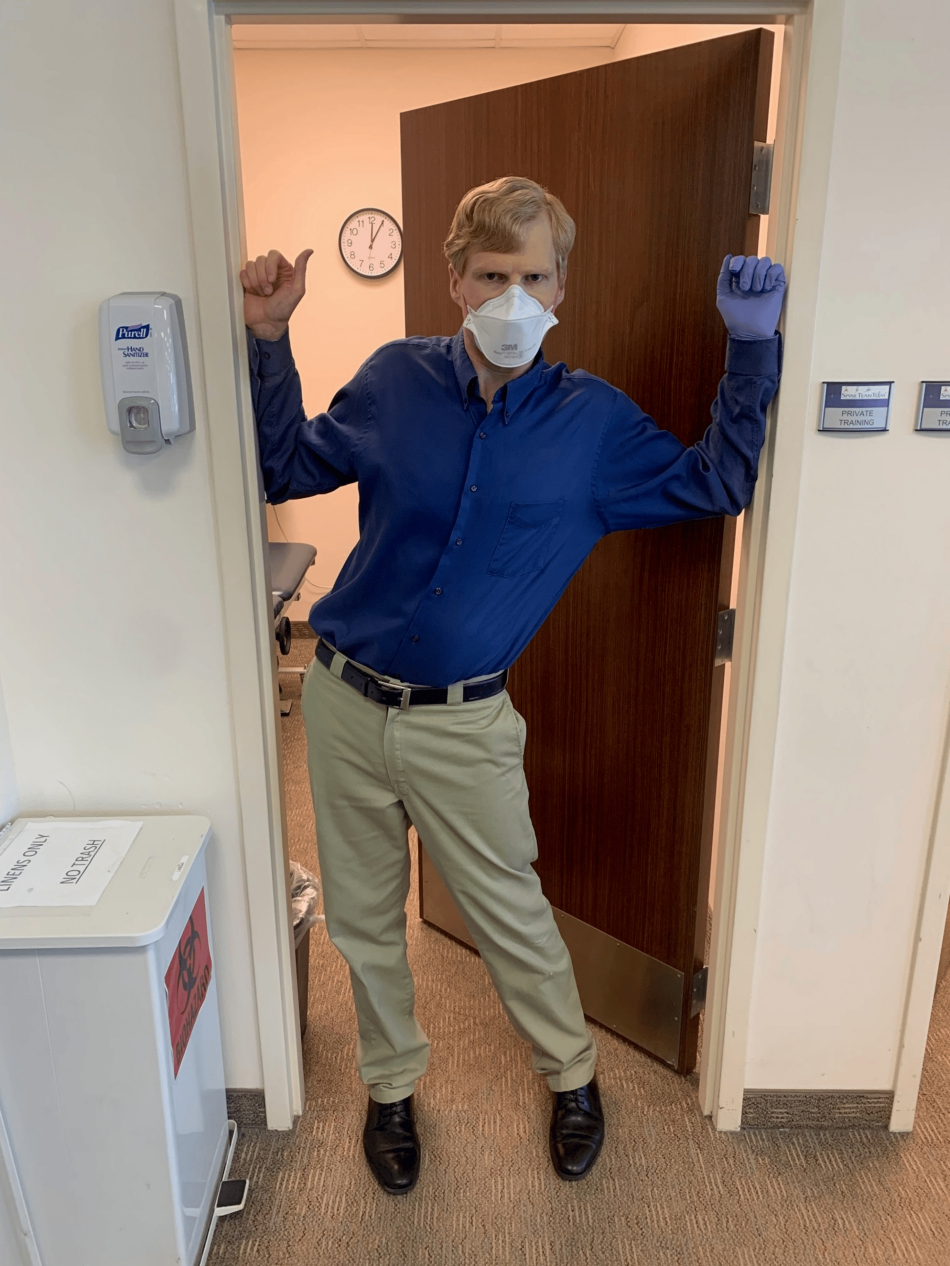
Left lumbar side gliding in a doorway. Representative image depicting the treating physical therapist.

Clinicians may wish to first have patients perform side gliding in a doorway (Figure 5) or free standing. As long as the response is not worse, then this author encourages performing shift correction with a better response to objective baselines and pain rating before progressing to a high repetition wall-assisted home program [20]. Clinicians are advised to utilize caution regarding patients receiving external force with side glides by family members or friends and ideally bring these individuals into the clinic to train them on the correct form of shift correction. Further research and systematic adverse event surveillance are needed to determine whether this is an isolated incident or a reproducible risk under specific circumstances.

The primary limitation of this case report is incomplete information. Medical records were unable to be obtained from both the orthopedic doctor and the second treating Physical Therapist. Additionally, medication usage by this patient is largely unknown. Moreover, this case report involved a lack of numeric quantification of lumbar AROM. Furthermore, immediately after this critical event, subjective and objective baselines, such as NPRS, strength, and AROM, were not adequately documented.

## Conclusions

Side gliding against a wall is a very safe and, at times, highly effective exercise. However, this case report observed a temporal association of acute hip pain after side gliding against a wall, in the context of the patient having performed around 160 repetitions per day of lumbar side gliding against a wall for the prior 18 days, having an untrained family member give this movement a push, and preexisting hip internal rotation weakness. Clinicians may wish to utilize shift correction to confirm both the safety and benefit of side gliding in a particular direction before teaching patients to perform side gliding against a wall. Beyond this, testing for internal hip rotation weakness may be wise because it is possible that an adverse event to the hip with side gliding may only be possible in the presence of hip internal rotation weakness. Moreover, clinicians are to utilize caution with a patient's family members or friends who wish to provide manual assistance with side gliding, ideally providing supervised instruction to these individuals. Furthermore, after an adverse event in the clinic, providers are advised to collect subjective and objective information such as NPRS, strength, and AROM. Additionally, after an adverse event, consider obtaining imaging as well as referring to an orthopedic physician for further evaluation.
